# *N*-[3-(Benzene­sulfonamido)­prop­yl]benzene­sulfonamide

**DOI:** 10.1107/S1600536811020150

**Published:** 2011-06-18

**Authors:** Tahir Ali Sheikh, Islam Ullah Khan, William T. A. Harrison, Ejaz

**Affiliations:** a Materials Chemistry Laboratory, Department of Chemistry, GC University, Lahore 54000, Pakistan; b Department of Chemistry, University of Aberdeen, Meston Walk, Aberdeen AB24 3UE, Scotland

## Abstract

In the title compound, C_15_H_18_N_2_O_4_S_2_, the dihedral angle between the aromatic rings is 71.8 (2)°. The conformation of the central N—C—C—C—N fragment is *gauche*–*gauche* [torsion angles = 72.5 (5) and 65.7 (5)°]. Both N atoms adopt pyramidal geometries. In the crystal, mol­ecules are linked by N—H⋯O hydrogen bonds, generating (001) sheets, and weak C—H⋯O inter­actions consolidate the packing.

## Related literature

For a related structure, see: Linden & Bienz (1999 ▶).
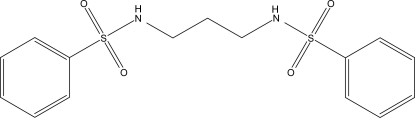


## Experimental

### 

#### Crystal data

C_15_H_18_N_2_O_4_S_2_
*M* *_r_* = 354.43
Orthorhombic, 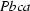

*a* = 9.2650 (13) Å
*b* = 16.402 (2) Å
*c* = 22.740 (3) Å
*V* = 3455.5 (8) Å^3^
*Z* = 8
Mo *K*α radiation
μ = 0.33 mm^−1^
*T* = 296 K
0.40 × 0.20 × 0.20 mm

#### Data collection

Bruker APEXII CCD diffractometer
13896 measured reflections
3393 independent reflections
1607 reflections with *I* > 2σ(*I*)
*R*_int_ = 0.091

#### Refinement

*R*[*F* ^2^ > 2σ(*F* ^2^)] = 0.069
*wR*(*F* ^2^) = 0.181
*S* = 1.03
3393 reflections
215 parameters
H atoms treated by a mixture of independent and constrained refinement
Δρ_max_ = 0.23 e Å^−3^
Δρ_min_ = −0.35 e Å^−3^

### 

Data collection: *APEX2* (Bruker, 2007 ▶); cell refinement: *SAINT* (Bruker, 2007 ▶); data reduction: *SAINT*; program(s) used to solve structure: *SHELXS97* (Sheldrick, 2008 ▶); program(s) used to refine structure: *SHELXL97* (Sheldrick, 2008 ▶); molecular graphics: *ORTEP-3* (Farrugia, 1997 ▶); software used to prepare material for publication: *SHELXL97*.

## Supplementary Material

Crystal structure: contains datablock(s) I, global. DOI: 10.1107/S1600536811020150/om2431sup1.cif

Structure factors: contains datablock(s) I. DOI: 10.1107/S1600536811020150/om2431Isup2.hkl

Supplementary material file. DOI: 10.1107/S1600536811020150/om2431Isup3.cml

Additional supplementary materials:  crystallographic information; 3D view; checkCIF report

## Figures and Tables

**Table 1:** Hydrogen-bond geometry (Å, °)

*D*—H⋯*A*	*D*—H	H⋯*A*	*D*⋯*A*	*D*—H⋯*A*
N1—H1⋯O4^i^	0.82 (4)	2.15 (5)	2.954 (6)	164 (4)
N2—H2⋯O3^ii^	0.74 (4)	2.15 (4)	2.836 (4)	154 (5)
C9—H9*B*⋯O4^iii^	0.97	2.51	3.430 (5)	158
C13—H13⋯O1^iv^	0.93	2.42	3.276 (8)	153

Symmetry codes: (i) 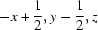; (ii) 
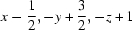
; (iii) 
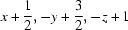
; (iv) 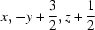.

**Fig. 1.**
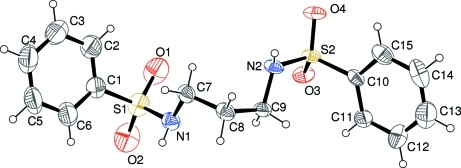
The molecular structure of (I) with displacement ellipsoids drawn at the 50% probability level.

## supplementary crystallographic information

### Comment

The title compound, (I), complements N-{4-[(benzenesulfonyl)amino]butyl}benzenesulfonamide, C_16_H_20_N_2_O_4_S_2_ (Linden & Bienz, 1999), (II), with a propyl chain in (I) replacing the butyl chain in (II).

In (I) (Fig. 1), the dihedral angle between the aromatic rings is 71.8 (2)°.  The conformation of the central N—C—C—C—N chain linking the two S atoms can be described as gauche–gauche in terms of the N1—C7—C8—C9 and C7—C8—C9—N2 torsion angles of 72.5 (5) and 65.7 (5)°, respectively.   Both N atoms in (I) are clearly in pyramidal coordination geometries, implying that the lone pairs on the N atoms are not conjugated with their adjacent benzene sulfonyl groups.  A similar situation was observed in (II).

In the crystal of (I), the molecules are linked by N—H···O hydrogen bonds (Table 1).  Considered separately, the N1 bond leads to [010] C(8) chains and the N2 bond generates [100] C(4) chains.  Both the acceptor O atoms are part of the same (atom S2) sulfonyl group: it is perhaps notable that these O atoms have significantly smaller U_eq_ values that the O atoms in the other (atom S1) sulfonyl group that do not accept a hydrogen bond.  Overall, (001) sheets arise from the N—H···O hydrogen bonds in (I) and weak C—H···O links consolidate the packing.

The complete molecule of (II) is generated by inversion symmetry and therefore the conformation of the central alkyl chain is all-trans and the dihedral angle between the aromatic rings is constrained to be zero by symmetry.

### Experimental

A mixture of 1,3-diaminoprpoane (0.0067 mol, 0.561 ml) and benzene sulfonyl chloride (0.0135 mol, 1.72 ml), was stirred in 15 ml of distilled water, while maintaining the pH of the reaction mixture at 9 using 3% sodium carbonate. The progress of the reaction was checked by TLC. On completion, the precipitate obtained was filtered, washed with water and finally dried.  Colourless blocks of (I) were grown from methanol by slow evaporation.

### Refinement

The N-bound H atoms were located in difference maps and their positions were freely refined with the constraint *U*_iso_(H) = 1.2*U*_eq_(N). The C-bound H atoms were placed at idealised positions and refined as riding with *U*_iso_(H) = 1.2*U*_eq_(C).

### Crystal data

**Table:**

C_15_H_18_N_2_O_4_S_2_	*F*(000) = 1488
*M**_r_* = 354.43	*D*_x_ = 1.363 Mg m^−^^3^
Orthorhombic, *P**b**c**a*	Mo *K*α radiation, λ = 0.71073 Å
Hall symbol: -P 2ac 2ab	Cell parameters from 2014 reflections
*a* = 9.2650 (13) Å	θ = 2.6–21.2°
*b* = 16.402 (2) Å	µ = 0.33 mm^−^^1^
*c* = 22.740 (3) Å	*T* = 296 K
*V* = 3455.5 (8) Å^3^	Prism, colourless
*Z* = 8	0.40 × 0.20 × 0.20 mm

### Data collection

**Table:**

Bruker APEXII CCD diffractometer	1607 reflections with *I* > 2σ(*I*)
Radiation source: fine-focus sealed tube	*R*_int_ = 0.091
graphite	θ_max_ = 26.0°, θ_min_ = 2.5°
ω scans	*h* = −5→11
13896 measured reflections	*k* = −18→20
3393 independent reflections	*l* = −28→27

### Refinement

**Table:**

Refinement on *F*^2^	Primary atom site location: structure-invariant direct methods
Least-squares matrix: full	Secondary atom site location: difference Fourier map
*R*[*F*^2^ > 2σ(*F*^2^)] = 0.069	Hydrogen site location: difmap (N-H) and geom (C-H)
*wR*(*F*^2^) = 0.181	H atoms treated by a mixture of independent and constrained refinement
*S* = 1.03	*w* = 1/[σ^2^(*F*_o_^2^) + (0.0722*P*)^2^ + 0.3591*P*] where *P* = (*F*_o_^2^ + 2*F*_c_^2^)/3
3393 reflections	(Δ/σ)_max_ < 0.001
215 parameters	Δρ_max_ = 0.23 e Å^−^^3^
0 restraints	Δρ_min_ = −0.35 e Å^−^^3^

### Special details

**Table:**

Geometry. All esds (except the esd in the dihedral angle between two l.s. planes) are estimated using the full covariance matrix. The cell esds are taken into account individually in the estimation of esds in distances, angles and torsion angles; correlations between esds in cell parameters are only used when they are defined by crystal symmetry. An approximate (isotropic) treatment of cell esds is used for estimating esds involving l.s. planes.
Refinement. Refinement of F^2^ against ALL reflections. The weighted R-factor wR and goodness of fit S are based on F^2^, conventional R-factors R are based on F, with F set to zero for negative F^2^. The threshold expression of F^2^ > 2sigma(F^2^) is used only for calculating R-factors(gt) etc. and is not relevant to the choice of reflections for refinement. R-factors based on F^2^ are statistically about twice as large as those based on F, and R- factors based on ALL data will be even larger.

### Fractional atomic coordinates and isotropic or equivalent isotropic displacement parameters (Å^2^)

**Table:**

	*x*	*y*	*z*	*U*_iso_*/*U*_eq_	
C1	0.1983 (7)	0.4365 (4)	0.3462 (2)	0.0749 (16)	
C2	0.0703 (9)	0.4669 (4)	0.3255 (3)	0.102 (2)	
H2A	0.0580	0.5230	0.3219	0.123*	
C3	−0.0417 (9)	0.4144 (6)	0.3098 (3)	0.124 (3)	
H3A	−0.1276	0.4354	0.2950	0.148*	
C4	−0.0255 (10)	0.3346 (5)	0.3158 (3)	0.118 (3)	
H4A	−0.1004	0.2997	0.3053	0.141*	
C5	0.0988 (12)	0.3034 (4)	0.3372 (3)	0.118 (3)	
H5	0.1090	0.2473	0.3414	0.142*	
C6	0.2110 (8)	0.3546 (4)	0.3528 (3)	0.102 (2)	
H6	0.2958	0.3327	0.3679	0.123*	
C7	0.1906 (5)	0.5379 (3)	0.4664 (2)	0.0624 (13)	
H7A	0.1191	0.4948	0.4645	0.075*	
H7B	0.1539	0.5845	0.4447	0.075*	
C8	0.2153 (5)	0.5616 (3)	0.5296 (2)	0.0603 (13)	
H8A	0.1224	0.5666	0.5489	0.072*	
H8B	0.2677	0.5179	0.5489	0.072*	
C9	0.2973 (4)	0.6398 (2)	0.5381 (2)	0.0556 (12)	
H9A	0.3180	0.6476	0.5795	0.067*	
H9B	0.3883	0.6371	0.5171	0.067*	
C10	0.3019 (4)	0.8225 (2)	0.5942 (2)	0.0490 (11)	
C11	0.4299 (5)	0.7968 (3)	0.6201 (2)	0.0680 (14)	
H11	0.5002	0.7705	0.5980	0.082*	
C12	0.4507 (8)	0.8108 (4)	0.6785 (3)	0.096 (2)	
H12	0.5355	0.7930	0.6963	0.116*	
C13	0.3495 (10)	0.8504 (5)	0.7115 (3)	0.106 (2)	
H13	0.3650	0.8589	0.7514	0.128*	
C14	0.2258 (8)	0.8775 (3)	0.6857 (3)	0.095 (2)	
H14	0.1585	0.9061	0.7079	0.114*	
C15	0.1992 (6)	0.8627 (3)	0.6261 (3)	0.0738 (15)	
H15	0.1136	0.8798	0.6087	0.089*	
S1	0.33690 (18)	0.50195 (11)	0.36927 (6)	0.0851 (5)	
S2	0.27067 (10)	0.79981 (7)	0.52013 (5)	0.0495 (4)	
N1	0.3261 (4)	0.5097 (3)	0.43942 (19)	0.0622 (12)	
H1	0.349 (5)	0.466 (3)	0.455 (2)	0.064 (17)*	
N2	0.2115 (3)	0.7085 (2)	0.51605 (18)	0.0547 (10)	
H2	0.135 (5)	0.708 (3)	0.525 (2)	0.066*	
O1	0.3051 (5)	0.5811 (3)	0.34561 (18)	0.1226 (17)	
O2	0.4721 (5)	0.4642 (3)	0.35702 (17)	0.1228 (17)	
O3	0.4064 (3)	0.8029 (2)	0.49056 (13)	0.0654 (9)	
O4	0.1556 (3)	0.85005 (18)	0.49911 (14)	0.0637 (9)	

### Atomic displacement parameters (Å^2^)

**Table:**

	*U*^11^	*U*^22^	*U*^33^	*U*^12^	*U*^13^	*U*^23^
C1	0.106 (5)	0.064 (4)	0.054 (3)	−0.007 (3)	0.002 (3)	0.011 (3)
C2	0.140 (6)	0.064 (4)	0.104 (5)	−0.017 (4)	−0.029 (5)	0.018 (3)
C3	0.137 (7)	0.110 (7)	0.125 (6)	−0.012 (5)	−0.049 (5)	−0.002 (5)
C4	0.170 (8)	0.086 (6)	0.097 (5)	−0.031 (6)	−0.025 (5)	−0.008 (4)
C5	0.202 (9)	0.059 (5)	0.093 (5)	−0.010 (6)	−0.022 (6)	−0.010 (4)
C6	0.144 (6)	0.072 (5)	0.090 (4)	0.001 (5)	−0.016 (4)	−0.009 (4)
C7	0.051 (3)	0.048 (3)	0.088 (4)	−0.005 (2)	−0.003 (3)	−0.006 (3)
C8	0.053 (3)	0.045 (3)	0.083 (4)	−0.003 (2)	0.002 (3)	0.005 (2)
C9	0.044 (2)	0.046 (3)	0.077 (3)	0.005 (2)	−0.013 (2)	0.000 (2)
C10	0.045 (3)	0.033 (2)	0.068 (3)	0.0005 (19)	0.009 (2)	0.000 (2)
C11	0.055 (3)	0.067 (4)	0.082 (4)	0.005 (3)	−0.008 (3)	−0.011 (3)
C12	0.103 (5)	0.098 (5)	0.088 (5)	−0.012 (4)	−0.021 (4)	−0.020 (4)
C13	0.144 (7)	0.089 (5)	0.086 (5)	−0.015 (5)	−0.001 (5)	−0.005 (4)
C14	0.122 (6)	0.063 (4)	0.099 (5)	−0.010 (4)	0.045 (5)	−0.017 (4)
C15	0.069 (4)	0.055 (4)	0.098 (4)	0.001 (3)	0.017 (3)	0.006 (3)
S1	0.0949 (12)	0.0843 (12)	0.0762 (10)	−0.0253 (10)	0.0100 (8)	0.0154 (9)
S2	0.0281 (5)	0.0490 (7)	0.0714 (8)	0.0022 (5)	0.0015 (5)	0.0115 (6)
N1	0.062 (3)	0.049 (3)	0.075 (3)	−0.004 (2)	−0.002 (2)	0.005 (2)
N2	0.0289 (17)	0.048 (2)	0.087 (3)	0.0049 (18)	−0.001 (2)	0.007 (2)
O1	0.176 (5)	0.095 (3)	0.098 (3)	−0.049 (3)	−0.024 (3)	0.050 (3)
O2	0.091 (3)	0.169 (5)	0.108 (3)	−0.015 (3)	0.045 (3)	−0.021 (3)
O3	0.0318 (15)	0.090 (2)	0.075 (2)	−0.0012 (16)	0.0080 (15)	0.0128 (18)
O4	0.0395 (16)	0.057 (2)	0.095 (2)	0.0091 (15)	−0.0078 (16)	0.0255 (17)

### Geometric parameters (Å, °)

**Table:**

C1—C6	1.357 (7)	C9—H9B	0.9700
C1—C2	1.370 (8)	C10—C15	1.368 (6)
C1—S1	1.754 (6)	C10—C11	1.390 (6)
C2—C3	1.395 (9)	C10—S2	1.748 (5)
C2—H2A	0.9300	C11—C12	1.362 (7)
C3—C4	1.325 (9)	C11—H11	0.9300
C3—H3A	0.9300	C12—C13	1.365 (9)
C4—C5	1.351 (9)	C12—H12	0.9300
C4—H4A	0.9300	C13—C14	1.362 (9)
C5—C6	1.382 (9)	C13—H13	0.9300
C5—H5	0.9300	C14—C15	1.398 (7)
C6—H6	0.9300	C14—H14	0.9300
C7—N1	1.471 (6)	C15—H15	0.9300
C7—C8	1.506 (6)	S1—O2	1.424 (4)
C7—H7A	0.9700	S1—O1	1.435 (4)
C7—H7B	0.9700	S1—N1	1.603 (4)
C8—C9	1.503 (6)	S2—O3	1.427 (3)
C8—H8A	0.9700	S2—O4	1.430 (3)
C8—H8B	0.9700	S2—N2	1.597 (4)
C9—N2	1.468 (5)	N1—H1	0.82 (4)
C9—H9A	0.9700	N2—H2	0.74 (4)
			
C6—C1—C2	118.3 (6)	C15—C10—C11	120.9 (5)
C6—C1—S1	120.7 (5)	C15—C10—S2	120.0 (4)
C2—C1—S1	120.9 (5)	C11—C10—S2	119.1 (4)
C1—C2—C3	120.4 (6)	C12—C11—C10	118.9 (5)
C1—C2—H2A	119.8	C12—C11—H11	120.5
C3—C2—H2A	119.8	C10—C11—H11	120.5
C4—C3—C2	120.0 (8)	C11—C12—C13	121.2 (6)
C4—C3—H3A	120.0	C11—C12—H12	119.4
C2—C3—H3A	120.0	C13—C12—H12	119.4
C3—C4—C5	120.5 (8)	C14—C13—C12	119.8 (6)
C3—C4—H4A	119.7	C14—C13—H13	120.1
C5—C4—H4A	119.7	C12—C13—H13	120.1
C4—C5—C6	120.3 (7)	C13—C14—C15	120.6 (6)
C4—C5—H5	119.9	C13—C14—H14	119.7
C6—C5—H5	119.9	C15—C14—H14	119.7
C1—C6—C5	120.5 (7)	C10—C15—C14	118.4 (5)
C1—C6—H6	119.7	C10—C15—H15	120.8
C5—C6—H6	119.7	C14—C15—H15	120.8
N1—C7—C8	110.4 (4)	O2—S1—O1	120.0 (3)
N1—C7—H7A	109.6	O2—S1—N1	106.5 (3)
C8—C7—H7A	109.6	O1—S1—N1	106.8 (3)
N1—C7—H7B	109.6	O2—S1—C1	108.6 (3)
C8—C7—H7B	109.6	O1—S1—C1	106.9 (3)
H7A—C7—H7B	108.1	N1—S1—C1	107.4 (2)
C9—C8—C7	114.8 (4)	O3—S2—O4	118.66 (18)
C9—C8—H8A	108.6	O3—S2—N2	107.9 (2)
C7—C8—H8A	108.6	O4—S2—N2	105.36 (18)
C9—C8—H8B	108.6	O3—S2—C10	107.48 (19)
C7—C8—H8B	108.6	O4—S2—C10	108.8 (2)
H8A—C8—H8B	107.6	N2—S2—C10	108.2 (2)
N2—C9—C8	109.8 (3)	C7—N1—S1	119.5 (4)
N2—C9—H9A	109.7	C7—N1—H1	108 (3)
C8—C9—H9A	109.7	S1—N1—H1	110 (3)
N2—C9—H9B	109.7	C9—N2—S2	121.0 (3)
C8—C9—H9B	109.7	C9—N2—H2	115 (4)
H9A—C9—H9B	108.2	S2—N2—H2	109 (4)
			
C6—C1—C2—C3	−2.1 (9)	C2—C1—S1—O2	−147.7 (5)
S1—C1—C2—C3	−177.1 (5)	C6—C1—S1—O1	168.2 (5)
C1—C2—C3—C4	1.2 (11)	C2—C1—S1—O1	−16.9 (6)
C2—C3—C4—C5	0.0 (12)	C6—C1—S1—N1	−77.5 (5)
C3—C4—C5—C6	−0.2 (12)	C2—C1—S1—N1	97.4 (5)
C2—C1—C6—C5	1.9 (9)	C15—C10—S2—O3	147.5 (4)
S1—C1—C6—C5	176.9 (5)	C11—C10—S2—O3	−34.5 (4)
C4—C5—C6—C1	−0.8 (10)	C15—C10—S2—O4	17.8 (4)
N1—C7—C8—C9	72.5 (5)	C11—C10—S2—O4	−164.2 (3)
C7—C8—C9—N2	65.7 (5)	C15—C10—S2—N2	−96.2 (4)
C15—C10—C11—C12	1.4 (7)	C11—C10—S2—N2	81.8 (4)
S2—C10—C11—C12	−176.6 (4)	C8—C7—N1—S1	−165.2 (3)
C10—C11—C12—C13	−1.0 (9)	O2—S1—N1—C7	−173.0 (4)
C11—C12—C13—C14	−0.8 (10)	O1—S1—N1—C7	57.7 (4)
C12—C13—C14—C15	2.2 (10)	C1—S1—N1—C7	−56.7 (4)
C11—C10—C15—C14	0.0 (7)	C8—C9—N2—S2	179.2 (3)
S2—C10—C15—C14	178.0 (4)	O3—S2—N2—C9	56.7 (4)
C13—C14—C15—C10	−1.8 (8)	O4—S2—N2—C9	−175.6 (3)
C6—C1—S1—O2	37.4 (5)	C10—S2—N2—C9	−59.3 (4)

### Hydrogen-bond geometry (Å, °)

**Table:**

*D*—H···*A*	*D*—H	H···*A*	*D*···*A*	*D*—H···*A*
N1—H1···O4^i^	0.82 (4)	2.15 (5)	2.954 (6)	164 (4)
N2—H2···O3^ii^	0.74 (4)	2.15 (4)	2.836 (4)	154 (5)
C9—H9B···O4^iii^	0.97	2.51	3.430 (5)	158
C13—H13···O1^iv^	0.93	2.42	3.276 (8)	153

Symmetry codes: (i) −*x*+1/2, *y*−1/2, *z*; (ii) *x*−1/2, −*y*+3/2, −*z*+1; (iii) *x*+1/2, −*y*+3/2, −*z*+1; (iv) *x*, −*y*+3/2, *z*+1/2.

## References

[bb1] Bruker (2007). *APEX2* and *SAINT* Bruker AXS Inc., Madison, Wisconsin, USA.

[bb2] Farrugia, L. J. (1997). *J. Appl. Cryst.* **30**, 565.

[bb3] Linden, A. & Bienz, S. (1999). *Acta Cryst.* C**55** IUC9900046.

[bb4] Sheldrick, G. M. (2008). *Acta Cryst.* A**64**, 112–122. 10.1107/S0108767307043930 18156677

